# The quality of maternal-fetal and newborn care services in Jordan: a qualitative focus group study

**DOI:** 10.1186/s12913-019-4232-9

**Published:** 2019-06-26

**Authors:** Mohammad S. Alyahya, Yousef S. Khader, Anwar Batieha, Majed Asad

**Affiliations:** 1 Department of Health Management and Policy, Faculty of Medicine, Jordan University of Science and Technology, P.O. Box: 3030, Irbid, 22110 Jordan; 2 Department of Public Health and Community Medicine, Faculty of Medicine, Jordan University of Science and Technology, Irbid, 22110 Jordan; 3 Jordan Ministry of Health, Directorate of non-communicable diseases, Amman, Jordan

**Keywords:** Antenatal care (ANC), Postnatal care (PNC), Continuity of care, Care coordination, Maternal quality, Child care quality, Care collaboration, Newborn care

## Abstract

**Background:**

The antenatal, intrapartum, and postnatal periods are considered high-risk periods for the health of mothers and their newborns. Although the current utilization rate of some maternal and child care services in Jordan is encouraging, detailed information about the quality of these services is limited. Therefore, this study aimed to explore the quality of maternal-fetal and newborn antenatal care (ANC), delivery, and postnatal care (PNC) services in Jordan.

**Methods:**

We conducted 12 focus group discussions (FGDs) with pregnant and postpartum women who attended maternal-child care services in three major hospitals in Jordan. All FGDs were recorded and transcribed verbatim. An inductive thematic analysis approach was used to identify themes and subthemes.

**Results:**

The content analysis of the FGDs revealed a consensus among the discussants regarding the importance of ANC and PNC services for the health of mothers and their newborns. However, the participating women viewed ANC to be much more important than PNC. With regards to the choice between public and private antenatal care services, some of the discussants were disposed towards the private sector. Reasons for this included longer consultation time, a higher quality of services, better interpersonal and communication skills of healthcare providers, better treatment, more advanced equipment and devices, availability of female obstetricians, and more flexible appointment times. These women only perceived public hospital services to be necessary in cases of pregnancy-related complications and labor, as the costs of private sector services in such cases are too high. The findings also revealed that mothers usually only seek PNC services to check up on their newborn’s health and not their own.

**Conclusion:**

Visiting private ANC clinics throughout pregnancy while giving birth in public facilities leads to the discontinuity and fragmentation in maternal-fetal and child healthcare services. To address this fragmentation, healthcare systems are proposed to establish interprofessional teamwork that requires different healthcare providers with complementary skills and practices in both public and private settings to work co-operatively and collectively. Investment in new technologies and interventions which enhance coordination and collaboration between public and private healthcare settings is necessary for the provision of non-traditional maternal healthcare.

**Electronic supplementary material:**

The online version of this article (10.1186/s12913-019-4232-9) contains supplementary material, which is available to authorized users.

## Background

Pregnancy, childbirth, and soon after childbirth are considered high-risk periods for the health of mothers and their newborns, especially in developing countries. Approximately 303,000 women die each year during pregnancy or the postpartum period. The vast majority (99%) of maternal deaths occur in developing countries, with most of these deaths being preventable [1–3]. In 2015, an estimated 2.6 million neonatal deaths and 2.1 million stillbirths were reported [4].

In low- and middle-income countries (LMICs), progress in addressing preventable maternal and newborn deaths and stillbirths depends on the improvement of the quality of maternal-fetal and newborn care throughout the continuum of care [5–7]. Therefore, it is vital that women and their newborn babies have access to high quality maternal care that is adequately equipped and staffed [8–10]. The antenatal care (ANC) guidelines and interventions can reduce maternal and perinatal mortality and morbidity through early diagnosis and management of pregnancy-related complications. They may also help in the identification of women who are at a high risk of developing delivery-related complications or having a premature birth. This ensures appropriate referral to secondary or specialist care. Several health prevention and promotion interventions take place during ANC visits, such as iron and folic acid supplementation and detection and investigation of hypertensive diseases of pregnancy [8, 11, 12]. During these visits, women are also encouraged to utilize other maternal services. This is particularly important given that women who attend ANC are more likely to use health facility delivery and postnatal (PNC) services [13–15].

The first 42 days are considered a critical period for the health of mothers and their infants. In this sense, PNC services have been shown to be effective in reducing infant mortality, as they enable healthcare workers to identify post-delivery complications and to provide timely treatment [16–18].

The primary components of maternal and child health services in Jordan include antenatal, delivery, and postnatal care, as well as vaccination coverage and treatment coverage of common childhood illnesses. During the antenatal period, women are recommended to receive two doses of the tetanus toxoid vaccine, as well as sufficient iron and folic acid supplements to prevent and/or treat anemia during pregnancy. Also, regular blood pressure checkups and tests to identify possible antenatal complications are considered a vital part of ANC services in Jordan. Pregnant women are also encouraged to carry out urine and blood tests on a regular basis in order to detect possible complications. They are also encouraged to attend antenatal visits on a monthly basis until the 28th gestational week, fortnightly until the 36th gestational week, and then weekly until the 40th week, resulting in an average of 12–13 visits during the course of pregnancy. With regards to intranatal care, the vast majority of pregnant women in Jordan give birth under the supervision of a trained health professional. Similarly, postnatal care includes postnatal checkups directly after delivery and before leaving the hospital, as well as a two-day postnatal checkup. However, the latter service is less accessed. As for child health services, a full program of vaccinations, recommended by the Jordanian Ministry of Health (MoH), [i.e. a vaccination against tuberculosis- Bacille Calmette Guerin (BCG)-, three doses each of the DPT (diphtheria, pertussis and tetanus) and polio vaccines, and measles-mumps-rubella (MMR) by the age of 12 months] is a requirement for the child immunization card that entitles children to enter school. When a child is registered at a Maternal and Child Health Center (MCHC), he/she receives a health card that shows the height and weight and the vaccinations he/she has received since birth [19, 20].

Although ANC coverage in Jordan is relatively satisfactory, in that almost 79% of pregnant women have at least seven ANC visits [20], less information is available on PNC coverage. A national survey revealed that the rate of PNC utilization within 48 h of delivery among Jordanian women is 83.4% [20]. However, a high utilization rate of maternal and child healthcare services does not necessarily reflect a high quality of care.

In Jordan, the maternal mortality rate is 19 per 100,000 live births, the neonatal mortality rate is 15 per 1000 live births, and the mortality rate of babies born at or after 24 weeks of gestation is 10.6 per 1000 births [21–23]. In a recent study, Khader et al. [21] reported that about 35% of stillbirths in Jordan are potentially preventable and that 30.3% can be avoided with optimal care. Antenatal and postnatal care services in Jordan are provided to women at different levels by obstetricians and midwives in both public and private healthcare settings. Available midwifery services include antenatal, postnatal, delivery, birth spacing, neonate and child health, breastfeeding, and immunization services in primary health centers and maternity hospitals in Jordan [24]. With regards to labor, health care policies promote hospital births and do not encourage home births. Despite the fact that 99.7% of Jordanian women undergo institutional delivery by skilled birth attendants [20], the majority of women (96%) prefer to receive ANC from a doctor, with only 3% of women seeking ANC from a midwife or a nurse [25]. There are several challenges related to nursing and midwifery practice in Jordan. These challenges include traditional nursing practice that does not reflect holistic care, lack of nursing practice quality, inadequate consideration given to primary health care services, high turnover rate of staff, and most importantly, severe shortage of midwives [24, 26].

Although the current utilization rate of ANC is encouraging, detailed information about the quality of maternal-fetal and child healthcare services is limited. However, most of the available literature about evaluating the quality of maternal and newborn health care services in Jordan have either used a quantitative design [26] or focused on the views of healthcare professionals when collecting data. Qualitative data on this topic, especially data which reflects the perceptions and experiences of the women who receive such services, is limited. Qualitative data provides a deeper and richer insight into the social norms, behaviors, and challenges that might affect Jordanian women’s ability to take optimal advantage of the available maternal and newborn healthcare services. Understanding the quality of maternal healthcare services, including the barriers and obstacles to the access of such services, requires exploring the social reality as felt or lived by the discussants [27, 28]. This allows for the full exploration of the discussants’ specific needs and fully captures the cultural and social factors that may influence their choices. Capturing such sensitive data about the beliefs, values, feelings, and motivations that inform certain behaviors [27] whilst trying to preserve context [29] is not usually possible with quantitative data collection methods that use structured tools/instruments for data collection.

Therefore, the current study aimed to explore the quality of maternal-fetal and newborn care in ANC, delivery, and PNC services from the perspectives of Jordanian women in order to better understand the gaps in the completeness of maternity and child care in Jordan.

## Methods

### Design and settings

This qualitative study was conducted as part of an assessment phase of a large-scale implementation research aimed at exploring surveillance and registration of perinatal deaths in Jordan. Since an open and broad methodological approach with no predefined specific issues and assumptions was taken, a large amount of empirical data was generated through 16 focus group discussions (FGDs) with healthcare workers and women. The FGDs with the health workers focused on the reality, challenges, and suggestions regarding the registration and documentation of perinatal deaths and the causes of death [30–32]. Meanwhile, the FGDs with the women focused on their experiences, perceptions, needs, and satisfaction towards maternal-fetal and child care services in Jordan, as well as the registration process of neonatal deaths and stillbirths. A total of 12 FGDs with women who attended three selected hospitals during the study periods were conducted. Four to five discussants were included in each group. The participant sociodemographic characteristics are presented in Table 1. All of the participating hospitals, two of which are maternal and pediatrics hospitals, are public, serve a large segment of the population, and are located in different geographical areas (North East, South, and North).

**Table 1:** Participant characteristics

FG1	Age (years)	Number of Children	Pregnancy status	Type of Visit
Hospital A.				
1	≥ 40	2–3	In the eighth month	ANC
2	32–39	≤ 1	In the eighth month	ANC
3	≥ 40	≥ 6	–	PNC
4	≥ 40	≤ 1	In the ninth month	ANC
5	≥ 40	2–3	In the fourth month	ANC
FG2				
1	24–31	≤ 1 (1 stillbirth)	In the eighth month	ANC
2	32–39	4–5	–	PNC
3	≥ 40	2–3	–	PNC
4	24–31	≤ 1 (1 stillbirth)	–	PNC
5	≤ 23	4–5	In the fifth month	ANC
FG3				
1	24–31	4–5	–	PNC
2	24–31	4–5	–	PNC
3	≥ 40	≤ 1	In the ninth month	ANC
4	24–31	2–3	In the seventh month	ANC
FG4				
1	32–39	≥ 6	In the sixth month	ANC
2	≥ 40	4–5	–	PNC
3	≤ 23	≤ 1	In the ninth month	ANC
4	24–31	2–3	In the fifth month	ANC
Hospital B.				
FG1	Age (years)	Number of Children	Pregnancy status	Type of Visit
1	32–39	4–5	In the ninth month	ANC
2	24–31	2–3	–	PNC
3	≤ 23	2–3	In the fifth month	ANC
4	24–31	2–3	In the ninth month	ANC
5	24–31	4–5	–	PNC
FG2				
1	24–31	2–3	–	PNC
2	24–31	≤ 1	In the ninth month	ANC
3	24–31	4–5	In the sixth month	ANC
4	24–31	≥ 6	–	PNC
FG3				
1	≤ 23	2–3	In the ninth month	ANC
2	24–31	2–3	In the eighth month	ANC
3	≤ 23	≤ 1	–	PNC
4	≤ 23	2–3	–	PNC
FG4				
1	32–39	≥ 6	In the eighth month	ANC
2	32–39	2–3	–	PNC
3	≤ 23	≤ 1	–	PNC
4	≤ 23	2–3	In the ninth month	ANC
Hospital C.				
FG1	Age (years)	Number of Children	Pregnancy status	Type of Visit
1	≤ 23	≤ 1	In the ninth month	ANC
2	≤ 23	≤ 1	In the fifth month	ANC
3	32–39	2–3	In the eighth month	ANC
4	32–39	2–3	–	PNC
FG2				
1	32–39	≥ 6	In the seventh month	ANC
2	≤ 23	≤ 1	In the ninth month	ANC
3	32–39	4–5	In the eighth month	ANC
4	≤ 23	≤ 1	–	PNC
5	≤ 23	≤ 1	–	PNC
FG3				
1	24–31	≤ 1	In the ninth month	ANC
2	24–31	≤ 1	In the seventh month	ANC
3	24–31	≤ 1	–	PNC
4	24–31	4–5	–	PNC
FG4				
1	32–39	2–3	In the eighth month	ANC
2	32–39	4–5	–	PNC
3	≤ 23	≤ 1	–	PNC
4	24–31	2–3	In the ninth month	ANC

### Sampling and participant recruitment

Pregnant and postpartum women who were receiving maternal-child care services at the selected hospitals were approached for inclusion. Initially, the research team scheduled a meeting with the head nurse of the maternal and child unit at each of the selected hospitals and explained the purpose of the study and the data collection procedure. The head nurses then informed pregnant women and mothers about the study and invited them to participate. They also provided women with the contact details of two female research members for any further questions or concerns. Then, the head nurse at each hospital scheduled the focus group sessions based on the participating women’s availability and convenience. After informed consent was obtained, a total of 52 women who attended the selected antenatal or postnatal care clinics were included in the study.

### Data collection

The focus group discussions were carried out over a period of five months in 2018. Two doctoral degree-level female interviewers/facilitators and two female note-takers were trained for the purpose of data collection. The number of discussants in each group varied between four to five women. The FGDs were held using interview guidelines which had been developed in English and then translated into Arabic by the principal investigator (PI) of the project (Additional file 1). After subsequent discussions, the guidelines have previously been approved by the technical committee of the project, which included senior healthcare professionals, senior academics, and researchers.

As part of the data collection process, the note-taker recorded field notes of the participants’ non-verbal expressions and reflections during the discussion. All FGDs were conducted in the local Arabic dialect and were digitally recorded, with the permission of the discussants.

### Ethical considerations

Ethical approval was sought and obtained from the Institutional Research Committee of Jordan University of Science and Technology and from the Institutional Research Committee of the Ministry of Health. The facilitators assured the participants that participation in the study was entirely voluntary and that all data, including the audio records, notes, and transcripts, would be kept in a secure, locked location (in locked filing cabinets in a secure office and/or on a password-protected computer). Similarly, the participants were informed that if participation caused them any stress or discomfort at any point during the study, they had the right to withdraw from the study without any negative consequences or penalties.

### Data analysis

All FGDs were fully transcribed and then checked for accuracy by the project team who attended the group discussions. All manuscripts were carefully read and a codebook was developed using a thematic inductive approach [33]. To enhance trustworthiness and credibility, two researchers independently read all of the manuscripts, including the interview transcripts and field notes, line-by-line several times. Then, the researchers read and compared both the transcripts and the field notes for each focus group individually. Integrating the data obtained from the FGDs and the field notes enhanced the strength of the data analysis and increased the dependability of the findings. Sections that had similar meanings or shared similar ideas were then indexed with separate colors and codes to synthesize and confirm the themes and subthemes which emerged from the discussions and to ensure that the main areas of interest had been covered and verified by the participants. A consensus on the themes and categories was reached before a thematic map was created. The content analysis procedure was completed in the original language, Arabic, in order to conserve the credibility of the findings. Forward/backward translation of themes, sub-themes, and quotes into English was then undertaken to resolve incongruity and achieve conceptual equivalency.

## Results

The following themes and sub-themes emerged from data analysis. It is important to note that the discussants women from the three hospitals shared similar experiences and views of maternal-fetal and newborn care services.

### Perceived reasons for seeking antenatal care

There was a general consensus among the groups on the importance of ANC services. Among the discussants, the most common reasons for seeking ANC services were to find out and avoid fetal anomalies and abnormalities at an early stage of pregnancy; to make sure that the fetus is in good health; to receive comprehensive care and follow-up sessions; to perform lab tests if necessary; to receive multivitamins, iron/folic acid supplements, and medications; and finally, to receive routine maternal checkups, including checkups for gestational diabetes and preeclampsia, and thus avoid any related complications. One woman said:

*“I usually visit my private doctor to carry out some medical exams, to make sure that my baby is safe and healthy, and to check up on my health.” Hospital B, FG2*

Another woman added:

*“Yes, it’s important that I visit the clinic to avoid developing high blood pressure or diabetes during my pregnancy.” Hospital A, FG3.*

### Private versus public maternal and neonatal care services

Overall, it was found that most discussants preferred seeking private antenatal care services, whilst others found public sector care services to be satisfactory.

Discussants who preferred private sector services gave several reasons for this. One woman stated:

*“The doctors in the private sector show me a detailed scan of my baby and carry out comprehensive check-ups for me and my baby.” Hospital C, FG4*

One mentioned reason for the non-comprehensive care in public sector care services was the lack of gynecologists and obstetricians.

*“We only have one doctor serving hundreds of women in the clinic…. there should be at least two doctors serving in such clinics.” Hospital C, FG4.*

Another woman, who criticized the antenatal services provided at public hospitals, said:

*“You spend a whole day at the hospital and all you get is a one- to two-minute check-up on the ultrasound, and the doctor doesn’t even talk to you.” Hospital A, FG3.*

On the other hand, many discussants were satisfied with the antenatal services provided at public hospitals:

*“When I visit the public clinic, I always receive good services, such as monthly medications and regular check-ups of my blood pressure. The problem there is that you have to wait for too long. However, they do provide you with everything you and your baby need, such as laboratory tests, medications, and so on.” Hospital B, FG1.*

*“Every time I come to the public health center, they carry out all the necessary tests, such as blood pressure, diabetes, and so on. The only downside really are the long queues.” Hospital A, FG1.*

Another reason that encouraged some of the women to choose the private sector was that they believed that the multivitamins prescribed to them by private obstetricians were of higher efficacy than those available in public hospitals. Also, the women trusted laboratory results conducted in the private sector more than those conducted in public hospitals.

*“Sometimes, you carry out a urine analysis in a public hospital and receive results that don’t show any sort of infection, even though you’re sure that you have a Urinary Tract Infection. However, if you do the same test in a private clinic, you get a positive result. Visiting a private clinic is important if you want correct results.” Hospital C, FG2.*

Two other women confirmed this, saying:

*“Correct laboratory-test results are those you get from private laboratories. On the other hand, results that you get from public hospitals might be wrong due to the high demand on their labs … which could increase errors.” Hospital A, FG4.*

*“I usually go to a public hospital for my routine monthly visits, but if I have to do laboratory tests or if I have a concern, I go to a private doctor.” Hospital A, FG1.*

There were disagreements among the discussants on the levels of competency of obstetricians in the private and the public sectors. One woman was very disappointed by the advice given to her by an obstetrician in a public hospital, which turned out to be wrong as compared to that given to her by a private obstetrician:

*“With my first baby, I approached a private clinic and the doctor there told me that I had “placental calcification” and that I might have a premature delivery. The doctor prescribed me some medications to prevent the incompleteness of the baby’s lung. I went to a public hospital to double check, and the doctor there denied that I might have an early delivery. However, I did end up having an early delivery, and I thank god that I’d had that medication prescribed by the private obstetrician and that my baby was okay.” Hospital B, FG1.*

A woman, who was also a nurse working at the public hospital she accessed for her delivery, shared her sad story:

*“I was experiencing labor pain at midnight and I had a 2cm dilatation. The midwives rang the doctor in duty, but he didn’t show up. I had to approach him in his office personally, but he didn’t believe I was in labor because I was so calm. He told me that he would carry out a cesarean on me in the morning. Throughout the whole night, they didn’t put me on the monitoring machine to check the baby’s pulse. In the morning, I gave birth to a dead boy.” Hospital A, FG2*

Another woman added:

*“I need to be comforted and assured that mine and my baby’s health is being monitored and checked. In the public sector, obstetricians don’t view blood pressure and blood glucose checks as necessary. However, every time I visit a private clinic, they check my blood pressure and blood sugar.” Hospital A, FG4.*

On the other hand, other discussants viewed public sector healthcare professionals to be just as competent as those in the private sector. One woman said:

*“Honestly, during pregnancy, I prefer to go to the public health center, which is only a 30-minute walk from my house. They provide good services. Just as there are competent professionals in the private sector, the public sector has competent professionals too.” Hospital B, FG1.*

Other discussants also believed that some midwives in Maternal & Child Health Centers (MCHCs) lacked the experience and skills necessary to correctly recognize any health conditions in the woman or her fetus.

*“My neighbor was pregnant with twins, but the midwives in a public health center could not even recognize that.” Hospital A, FG2.*

Another reported reason which discouraged women from visiting MCHCs was the lack of equipment in these centers. Further, the discussants perceived the quality of the equipment and devices in private sector clinics to be better than that in the public sector.

*“The ultrasound machine in the private clinic I go to is way better than the ones in public hospitals and clinics … It’s more advanced.” Hospital C, FG1.*

*“I prefer private clinics because the equipment there is more accurate and advanced.” Hospital A, FG3.*

*“We want modern ultrasound machines which can produce 4D images because this can help identify any problems or deformities in the fetus. This equipment is available in private clinics but costs 50 JD to use.” Hospital B, FG1.*

Nonetheless, some women reported visiting the MCHC regularly during pregnancy because it was nearby to where they lived.

*“The MCHC is only a walking distance from my home, so I usually go there, regardless of what’s in there.” Hospital A, FG2.*

The limited working hours of MCHCs was cited as another barrier to seeking public antenatal healthcare services.

*“I go to a private doctor because he’s available at any time convenient to me, unlike the MCHC, which has limited working hours.” Hospital B, FG4.*

Another factor which encouraged women to seek private obstetricians was the interpersonal and communication skills of these obstetricians. Some of the discussants believed that private obstetricians were more attentive to their concerns than obstetricians in public hospitals.

*“I experienced bleeding during an early stage of my pregnancy and my private doctor told me that this was normal in my case and that there was nothing to be worried about. This made me feel optimistic, as she had given enough attention to my concerns.” Hospital A, FG4.*

Other discussants added that:

*“They (i.e. public sector doctors) don’t treat you as a human being. Rather, they treat you as a machine or a number.” Hospital B, FG3.*

*“They (i.e. public sector doctors) don’t like me to ask questions … If I do, they get irritated.” Hospital C, FG4.*

On the other hand, several discussants mentioned that the most common reason that they had visited a private obstetrician had been so that they could choose to be seen by a female doctor, which is not always possible in public hospitals and healthcare centers. This may be of particular importance for some women who would rather not be seen by a male doctor for cultural reasons.

*“When I first got pregnant, my husband took me to a public hospital but told me that he didn’t want a male doctor seeing me. The doctor turned out to be a male, and so I didn’t let him check up on me or my baby. I ended up going to a private female obstetrician. The next time I went to the public hospital there was a female doctor, so I had my check-up there.” Hospital C, FG1*

*“I sometimes go to a private female doctor because my husband refuses that I be seen by a male doctor, and there aren’t always female doctors available at the public hospital. In the private sector, you can choose which doctor you want. In my opinion, this is a very important issue in our society, especially given that my dad is also very religious and conservative. Therefore, the choice isn’t always based on the level of competence and quality of service.” Hospital A, FG2*

One drawback which made women hesitant to seek private gynecologists and obstetricians often was the high cost per visit.

*“I give the private sector a score of 8 out of 10; if it wasn’t for the high costs, I would give it a 10.” Hospital A, FG1*

*“I used to go to a private clinic during my pregnancy, but I no longer go because I can’t afford it anymore.” Hospital C, FG3*

Nonetheless, some discussants still visited private clinics despite the high costs. One woman said:

*“The fees of private clinicians are very high, and most women cannot afford it. However, many women still choose to go to private clinicians even though most of these women are medically insured in public hospitals and can access all public hospital services for free.” Hospital B, FG1*

Long waiting-time was another major reported obstacle which discouraged mothers from attending antenatal follow-up visits in public facilities.

*“I once came to the outpatient clinic in the hospital and had to wait for forty other women to be seen by the doctor before it was my turn.” Hospital C, FG3*

*“The problem there (at the MCHC) is that you have to wait for too long. However, they do provide you with everything you and your baby need, such as lab tests, medications, and so on.” Hospital B, FG1*

Many women usually only seek public hospitals during the final month of pregnancy, just so that they can open a file for the proposed delivery and have their baby in the public hospital in which they are insured. Although they believe that the delivery services provided in the private sector are much better than those provided in public hospitals, these women usually cannot afford having the baby in a private hospital due to the high cost.

Women who experience medical complications during pregnancy usually receive closer follow-up sessions and regular medical investigations in the public sector than those with normal pregnancies. Thus, these women prefer to visit public facilities because they are insured there and do not have to pay for all the costly services. Moreover, private clinics are not always fully equipped with facilities, equipment, and medical laboratories, and they do not have multidisciplinary healthcare professionals to provide a wide range of specialized care for pregnant women who experience medical problems. This could lead to the underdiagnoses of some medical conditions during pregnancy.

*“I visited a private doctor throughout the whole period of my pregnancy. However, no one told me that my blood was negative, and the doctor who I was checking up with hadn’t identified this. Also, I have a family history of diabetes and hypertension, but, unfortunately, the doctor didn’t pay enough attention to this. I ended up having a dead baby, and I blame the doctor for this.” Hospital C, FG4*

*“If you have regular check-ups at a public hospital or health center, it’s easier for the doctor to transfer you to the hospital or to another doctor if any complications occur during pregnancy.” Hospital B, FG2*

### Intrapartum care

There was a consensus among the discussants that the delivery care services in the public sector were suboptimal. The women cited several reasons for this, including a lack of trust in the healthcare professionals’ skills and abilities, privacy concerns, and less-centered care.

*“I have a phobia of public healthcare child delivery staff.” Hospital C, FG1.*

Other women added:

*“In public hospitals, you don’t get your own room, which means you have no privacy.” Hospital B, FG3.*

*“You know, I had my baby in a public hospital and was discharged immediately after I gave birth. The obstetrician there only saw me for a few minutes. My husband asked him about mine and my baby’s health, and he just kept saying “she’s fine and so is your baby” … Honestly, they don’t care about your emotions or about your family.” Hospital A, FG4.*

The majority of the discussants agreed that the pain during delivery had been unbearable. One young woman, who had just given birth to her first baby two months ago, shared her experience and described:

*“That pain was just horrible; I literally thought I wouldn’t make it to the end.”* (*Hospital B, FG3).*

Another woman with three children narrated:

*“I kept screaming and begging for some kind of pain relief, but unfortunately, no one listened to me … the midwife kept telling me carelessly, “you don’t need any pain killers” … and she ignored me and didn’t give me any reassurance.” (Hospital A, FG2).*

Our findings also revealed that the discussants had mixed perceptions and experiences regarding the responsible midwife during delivery. Some women agreed that the midwife had been helpful and supportive during the whole delivery period. A young woman with two children shared her positive experience and said:

*“With my last baby, the midwife was calm the whole time and held my hand whenever I had strong pain … she was very helpful … she kept reminding me to take deep breaths during contractions.” (Hospital C, FG4).*

On the other hand, other discussants shared negative experiences with midwives during labor. A mother of one child said:

*“The midwife was shouting at me the whole time … I was unhappy with the service and felt sad and disappointed … I just needed someone to tell me that everything was going to be fine.” (Hospital C, FG3).*

Another woman added:

*“Because of the overload on maternal hospitals, I guess, you don’t always get the chance to be seen by an obstetrician before delivery … the examination time for each patient just isn’t enough for the obstetrician to properly evaluate the mother and her baby.” (Hospital B, FG3).*

### Postnatal care

The majority of the discussants viewed postnatal care as being important for checking up on the newborn’s health, but not the mother’s. Most of the participating women had attended public postnatal clinics a week after giving birth to carry out certain tests, including lab tests, for their baby.

*“I visit the public postnatal clinic to check-up on my baby’s head circumference, weight, and height, and I also go to get contraceptives.” Hospital B, FG3.*

*“I mainly visit the MCHC for immunizations for my baby.” Hospital C, FG1.*

The discussants had sought postnatal care clinics weeks after delivery only to receive contraceptives or if they had noticed any abnormal changes to their baby’s health.

*“To be honest, I only went to the hospital after giving birth to my daughter to get the pills. Other than that, I didn’t follow up with them.” Hospital C, FG2*

*“If there’s a rash on my baby’s skin I immediately take him to a doctor who I can trust. Sometimes, I might even take him to more than one doctor to make sure he receives the correct diagnosis.” Hospital B, FG4*

In general, the discussants viewed PNC as being less important than ANC. They also reported not trusting the available postnatal care services.

*“You know, I don’t think I need to visit the doctor after delivery, unless there’s something critical … Many women prefer to stay at home after delivery, unless there’s an emergency situation.” Hospital A, FG4*

*“During my first pregnancy, I took stabilizers up until the seventh month, but I was surprised to later find out that there had been no need for them at all. After this, I came to the conclusion that you don’t have to do everything that the doctor asks you to do. After I experienced giving birth to a dead baby, I learned the lesson that doctors’ orders aren’t holy.” Hospital C, FG3*

The women perceived postnatal care services in the public sector to be better than antenatal care services.

*“I give the postnatal care services in public hospitals a score of 7 out of 10. They are better than what I received during my pregnancy.” Hospital C, FG3.*

However, some women still preferred seeking private clinics for PNC services. They were willing to pay the extra costs, as they could then expect to receive superior care.

*“In private clinics, even though I have to pay much more, at least I don’t have to wait so long to be seen by the doctor. Also, all my questions and concerns are addressed by my doctor, and this isn’t the case in public hospitals, which have very long queues.” Hospital B, FG2*

Finally, discussants strongly believed that the lack of coordination between ANC, intrapartum care, and PNC services is one of the main factors which may negatively impact the health of women and newborns.

*“If you ask me why I gave birth to a dead baby in my last pregnancy, I’ll tell you that one of the main causes was related to the disharmony between the public hospital and the private clinic which I had been visiting. I came to the hospital with labor pain, and the doctor didn’t know anything about me or my baby. I was too tired to explain to him that I had high blood pressure … My sister was with me, but she too didn’t know that I had high blood pressure.” Hospital C, FG2.*

## Discussion

This study has explored women’s experiences of ANC, intrapartum care, and PNC services in Jordan and investigated the gaps in the provision of these services. The content analysis of the FGDs revealed that all of the discussant women acknowledged the importance of both ANC and PNC services in ensuring the health and safety of women during pregnancy and of newborns during and after delivery. Nonetheless, the mothers perceived ANC to be much more important than PNC. Globally, women generally have positive attitudes towards ANC and PNC [34–36]. However, certain communities in some developing countries consider pregnancy and childbirth and some of their related complications to be part of a woman’s natural life events. Hence, they perceive maternal and child care services as being unimportant [34, 37, 38].

Several reasons for seeking antenatal care services were discussed by the participants. These reasons included monitoring fetal development and detecting fetal anomalies, receiving comprehensive care and follow-up sessions, carrying out lab tests, receiving medications and multivitamins, and avoiding pregnancy complications such as gestational diabetes and preeclampsia. According to the WHO, ANC provides a platform for many important interventions and healthcare functions, including counseling for a healthy lifestyle, screening and diagnosis, and prevention of disease [8]. There is robust evidence that adopting timely and appropriate evidence-based ANC practices reduces maternal mortality and improves newborn health outcomes [8, 12, 39–41].

Empirical evidence from developing countries, including Jordan, has shed light on the influence of socio-economic, demographic, residential, and cultural factors on the utilization of maternal-fetal and child healthcare services. Educated women and women of high economic status are more likely to utilize ANC services than uneducated women or women of low economic status [42–45]. A positive correlation has been found between the percentage of Jordanian women who attend a minimum of seven ANC visits and household income. In specific, mothers with higher education are more likely (84%) to access antenatal care from any trained personnel than uneducated women (55%). An association has also been found between a pregnant woman’s education level and the timing of her first ANC visit. Highly-educated women are more likely (88%) to have their initial ANC visit during the first trimester of pregnancy than uneducated women (71%). Meanwhile, insignificant differences in the use of ANC services have been found among Jordanian women from urban areas and women from rural areas [20]. As with regards to the utilization of PNC services, health status, mode of delivery, level of education, number of children, awareness of need, cost, and distance have been found to be the most common predictors [46–48]. The percentage of Jordanian women who seek PNC services within the first two days of delivery ranged from 68% among uneducated women to 87% among highly-educated women. It was also found that women who are older than 35 years of age are more likely (88%) to attend postnatal check-ups than younger women (77%) [20].

Our findings have also revealed that proximity of the health facility is one of the major impacting factors on the discussants’ choice of facility. Research on the impact of the proximity of maternal healthcare services/facilities or the time required to travel to these facilities on maternal and neonatal outcomes is inconclusive [49]. One study found that the odds of unplanned delivery out of hospital increase if the distance to the health facility is more than 35 km [50]. Similarly, a positive correlation between maternal mortality and distance to hospital was observed [51, 52]. Women who lived more than 35 km away from the hospital had a four times higher maternal mortality rate than women who lived within 5 km. However, no effect of distance to hospital was shown on indirect maternal or pregnancy-related mortality, suggesting a lack in the quality of care received [52]. Based on the discussants’ perceptions, proximity to the health facility needs to be accounted for when evaluating maternal healthcare services.

There was some disagreement among the discussants on the difference in competency levels between healthcare workers in private facilities and those in public facilities. However, many of the participating women stated that they preferred to seek ANC services in private sector clinics rather than public sector facilities for several reasons. Some of the reported reasons included longer consultation time, higher quality services, better interpersonal and communication skills of the healthcare providers, more advanced equipment and devices, constant availability of female doctors, and more flexible appointment times. These reasons discouraged many of the women from seeking public hospitals unless they were during the final month of pregnancy. Similarly, a qualitative study by Titaley et al. [34] found that free public ANC services were believed to be associated with a lower quality of both healthcare services and medications. Other studies conducted in developing countries showed that private health facilities are preferred to public facilities due to several reasons, including more approachable and more helpful staff, easier access, well-equipped clinics with modern equipment and devices, and more professional healthcare providers who treat patients with respect and in a friendly manner. The only negative issue found to be associated with private clinics was the high cost of services as compared to the free cost of public facilities [53, 54].

The participating women perceived public hospital health services to be necessary only in the case of pregnancy-related complications, as they provide comprehensive care. Not all women can afford to give birth to their babies in private hospitals, and so many women choose to receive ANC at a private clinic but give birth in a public facility. This can significantly impact the continuum of services and can increase the fragmentation in care. Medical insurance status and financial concerns were found to influence the utilization and continuity of maternal, neonatal, and child public care services [34, 55].

While positive attitudes towards PNC were noted among the discussants, the findings have revealed that women usually only seek healthcare settings after delivery to check on their infant’s health (i.e. routine tests and vaccines) rather than their own. The women reported seeking postnatal facilities only to receive contraceptives or in cases of serious health conditions. However, PNC is important as it is an opportunity to identify any complications after delivery and to provide preventive services. Such services include the provision of family planning advice to avoid pregnancy soon after giving birth and improve the health and survival of both the mother and her child [10, 56, 57]. In line with the study findings, previous research has also found that, despite the perceived importance of postnatal care, these services usually target the newborn and pay little attention to the mother, unless there is a serious condition related to maternal complications [35]. Thus, more efforts need to be exerted to communicate the potential benefits of PNC more effectively.

Despite the importance of the postpartum period as a transitional time for the mother, the baby, and the whole family, it is, unfortunately, the most neglected period in terms of maternal care services [56]. Less than 45% of mothers in developing countries receive full PNC services, with mothers from rural areas having the least access to services [58–62]. According to the latest national survey (2017–2018), 83.4% of Jordanian mothers receive PNC within 48 h of delivery [20]. Nonetheless, there is a lack of evidence on the utilization patterns and predictors of full PNC services in Jordan and other Arab countries. In a systematic review by Jongh et al. [63], the authors reported that, generally, poor attention is given to PNC in LMIC, which explains the near complete absence of studies evaluating the potential benefits of integrating PNC with ANC. This also implies sustained fragmentation of the continuum of maternal and child healthcare services [63].

There was a consensus among the participating women that, in the public sector, the quality of postnatal care is better than the quality of antenatal care. Despite this, the women preferred receiving post-delivery care in private clinics rather than in public settings. Space limitations and staff shortages were among the perceived barriers to the provision of private and confidential maternal care in public sector facilities, as women usually have to sit together and take turns in receiving health services. This makes it difficult for a woman or her partner to discuss any personal concerns or vulnerable issues with the doctor without being overheard [64].

The women believed that discontinuity and poor quality of maternal and child care could lead to maternal mortality and perinatal death. Bhutta et al. [7] showed that increased coverage and quality across the continuum of care by 2025, including preconception (before and between pregnancy), antenatal, intrapartum, and postnatal interventions, could prevent 71% of neonatal deaths, 33% of stillbirths, and 54% of maternal mortalities each year in the 75 high-burden countries that together incur more than 95% of maternal, neonatal, and child deaths. Another study which analyzed data from DHS surveys conducted in 20 countries in Africa found a large gap between the number of visits (contacts) and the interventions received by mothers and newborns during these visits (content). This gap was seen as a proxy of the quality of care provided during antenatal, intrapartum, and postnatal periods in all of the 20 countries [65].

### Implications for further improvement

Continuity of healthcare is one of the main principles of healthcare systems and is considered as an effective health strategy to improve maternal and child health services [66–68]. Yet, it has not been adequately implemented and studied in LMIC [68–70]. Continuity of care (COC) can be divided into interpersonal COC, which can be defined as the extent to which a woman encounters the same healthcare providers for maternity care, and informational COC, which is the extent to which clinical information is available to all healthcare providers involved in a woman’s care [71–73]. Other researchers add another type of COC, namely management COC. Management continuity is critically important in managing chronic or complex clinical conditions that require multidisciplinary health providers [74].

Maternity care services in Jordan have proved to be fragmented, with poor communication between private and public healthcare providers. Furthermore, poor continuity between antenatal care settings and intrapartum and postpartum settings has been noted. This care fragmentation is risky and can threaten the quality and safety of the healthcare rendered to women and babies, as low continuity of maternal care is thought to contribute to women’s dissatisfaction, information loss, conflicting advice, overuse of maternal and child interventions, or even medical errors [67, 75–77]. In order to establish a maternal system of COC, several approaches and strategies have been implemented successfully in different countries. The midwife-led continuity (MLC) model is one of the promising strategies that could improve the utilization and quality of healthcare services for mothers and newborns [78–80]. In the MLC model, a known and trusted midwife, or small group of midwives, supports and helps the woman during pregnancy, delivery, and the postnatal period to enable a healthy pregnancy and childbirth and appropriate parenting practices [8, 79]. However, in reorganizing existing maternal care services in order to implement the MLC model, ensuring the availability of competent, autonomous, skilled, and knowledgeable midwives is critical for the success of this model [80]. This leads us to emphasize the importance of enhancing inter-professional collaboration and the improving communication between all maternity care providers at different levels and in different settings [75].

The MLC approach has been implemented successfully in several countries around the world (both developed and developing). In 2013, Palestine implemented a new MLC model, and a recent study which evaluated this model showed that the number of antenatal and postnatal visits per woman have increased significantly. PNC visits for mothers and newborns, including home visits, have also increased substantially since the introduction of this new model. Some quality indicators related to facility-level outcomes have also improved, including increased continuity, better functioning referral to a higher level of care mechanisms, and increased postnatal home care visits [78].

Maternal quality of care encompasses many aspects other than the number and type of services provided. Strong interpersonal communication skills and good interaction between healthcare providers and women and their families is likely to improve women’s satisfaction with the maternity care experience [64, 80–82]. In 2016, the WHO issued a new global guideline for the routine ANC, a guideline which emphasizes person-centered health and well-being based on a human rights approach [8]. This informs the development of an individualized approach which places the mother and her family at the center of care and aims to respond to their needs and preferences in a humane and holistic manner. A family- and woman-centered care system that keeps women and their families informed and actively involved in care and views them as participants as well as beneficiaries can encourage continuity of care and improve maternal care quality across health systems [80, 82–84].

An integrated maternal health system with shared electronic medical records and mobile technology tools can also make a significant contribution to COC through the enhancement of coordination between different healthcare providers at different levels and the provision of tailored maternity services [85, 86]. Mobile health (m-health) can potentially enhance pregnant women’s engagement with and trust in health professionals’ skills and can improve women-provider interaction. The introduction of new technology has proved to make mothers feel heard [87]. This requires the adoption of a new structure and the development of new practices based on collaboration to achieve the concept of “collective action” [85, 86].

Several other models and strategies have been proposed and successfully implemented world-wide to improve the quality of maternal care. Still, every health system needs to choose a model that is best suited for its existing infrastructure, resources, and culture. We cannot claim that there is a single or a group of models or approaches which can fit any country or health system to better meet the changing needs of women, children, and families [86]. For these reasons, inexpensive, non-traditional methods of maternal care are being proposed, such as giving each woman her own case notes. These case notes may come in the form of a portable card that contains a summary of the woman’s notes and pregnancy history and progress, to be carried to each maternal visit. This portable notes card method was introduced in several countries to promote easy access to the woman’s medical record and to avoid the high administrative cost of keeping and retrieving health records in the traditional manner, especially in low income countries that have poor infrastructure and limited funds [88]. Accordingly, it was recommended by the WHO (2016) that each pregnant woman keep her own case notes card throughout pregnancy as a method of increasing continuity, enhancing the quality of maternal care, and improving the woman’s pregnancy experience [8].

The development of innovative health promotion programs which aim to raise awareness among women and other community members about the protective role of postnatal care services is needed. Recently, Shaban et al. [89] studied the feasibility of initiating postnatal home visitation in Jordan and found that postnatal home visits were well-received by the participating women. This new model would best meet the needs of mothers, especially mothers who live in isolated areas and mothers who face financial and cultural barriers which prevent them from visiting maternal care facilities for PNC [89]. One example of such cultural barriers is the belief among some families that new mothers should stay at home for 40 days after giving birth [56, 89, 90]. Finally, developing the continuum of care for maternal and child health services will need effective interventions, policy support, public-health planning, strengthening of healthcare systems, and systematic efforts to overcome operational management challenges, especially human resources management [70].

### Strengths and limitations

The current study has several strengths. A total of 12 FGDs were conducted with women from different geographical locations in Jordan to reach a saturation of themes, thereby ensuring the quality and completeness of the findings. The focus groups were conducted in private meeting rooms in which the participating women felt safe and comfortable in sharing their experiences and views. All FGDs were conducted by female researchers with extensive experience in conducting interviews and facilitating FGDs. The process of data analysis enhanced the strength of the study. Two researchers independently and carefully read all the interview transcripts and field notes and then compared both the transcripts and the field notes for each focus group individually in order to enhance trustworthiness and credibility. Incorporating the data obtained from the field notes and the data which emerged from the FGDs increased the trustworthiness of the findings. The analysis of all transcripts and quotes was conducted in Arabic to guarantee the credibility of the themes. Finally, this qualitative study is a vital part of an assessment phase of a large-scale implementation research which is proposed to help in building a surveillance and registration system of perinatal deaths in Jordan, taking into account women’s perceptions and experiences in this regard.

One limitation of the current study is that the discussants were not asked about all the major components of ANC. However, we were hoping that the discussants would mention such components during the focus group discussions, especially as the flexible and emerging nature of this qualitative design usually allows discussants to unleash issues the researcher does not necessarily ask them about. This was especially expected considering the fact that the discussants were the key informants who were expected to enrich the discussion by sharing their experiences and perceptions of maternal services. Another limitation of the study is that the researchers did not take into consideration the role of certain factors such as the age, educational level, and place of residence of the women and the impact of first time pregnancy on the women’s perceptions of ANC, intrapartum care, and PNC.

## Conclusions

Our findings reveal that women perceive both ANC and PNC services to be important in ensuring the health and safety of mothers during pregnancy and of newborns during and after delivery. Nonetheless, there was a consensus among the discussants that ANC is more important than PNC. PNC has not been adequately prioritized as a maternal and child healthcare service. In addition, women generally prefer maternal services provided in private clinics to those provided in the public sector, unless they face serious health problems. In such cases, most women are forced to seek the public sector due to the high cost of services and care in the private sector, especially labor services. This is alarming, especially given that the majority of women in Jordan are medically insured in public facilities and that ANC and PNC services are accessible and almost free of charge in the public sector. Our study highlights certain barriers that discourage Jordanian women from accessing healthcare services in the public sector, including lack of privacy, lack of confidence in the skills of the healthcare providers, availability of female obstetricians, and a perceived inadequate quality of services. This entails empowering healthcare workers with clinical, interpersonal, and communication skills; improving the infrastructure and quality of public maternal and child care services; and providing women-centered care based on dignity and respect.

Due to the fragmented nature of maternity services in Jordan, maternity care reform is proposed to establish interprofessional team-work that requires obstetricians, family medicine doctors, and midwives in both public and private settings to work co-operatively and collectively with complementary skills and practices. Finally, this study emphasizes that the continuity of care for pregnant mothers and their newborns requires efforts beyond the provision of traditional ANC and PNC services. Investment in new technologies and interventions which promote coordination between public and private healthcare providers, such as postnatal home visits and the midwife-led continuity model, is required for enhancing maternal healthcare.

## Additional file

Additional file 1: Focus Group Discussions Guidelines with women: Quality of Maternal-Fetal and Newborn Care Services. (DOCX 14 kb)

## Data Availability

The qualitative data collected for the study contains sensitive, potentially identifiable, personal information and so will not be available in its entirety. Requests for suitably anonymized sections of the data can be made to the corresponding author.

## Abbreviations

ANC: Antenatal Care
BCG: Bacille Calmette Guerin
COC: Continuity Of Care
DHS: Demographic and Health Surveys
DPT: Diphtheria, Pertussis and Tetanus
FGDs: Focus Group Discussions
LMICs: Low- And Middle-Income Countries
MCHCs: Maternal & Child Health Centres
MLC: Midwife-Led Continuity
MMR: Measles-Mumps-Rubella
MoH: Ministry of Health
PI: Principal Investigator
PNC: Postnatal Care
WHO: World Health Organization
