# Supplementary material for: The quality of maternal-fetal and newborn care services in Jordan: a qualitative focus group study
Source: BMC Health Serv Res. 2019 Jun 26;19:425. doi: 10.1186/s12913-019-4232-9 (PMC6595569; doi:10.1186/s12913-019-4232-9)
Supplement: Supplementary file 1 — Focus Group Discussions Guidelines with women: Quality of Maternal-Fetal and Newborn Care Services. (DOCX 14 kb) [file 12913_2019_4232_MOESM1_ESM.docx]

**Focus Group Discussions Guidelines with women: Quality of Maternal-Fetal and Newborn Care Services**

We are conducting this research project to explore the quality of maternal-fetal and neonatal care services in Jordan. Your opinions would be very important for providing data on quality indicators to determine which intervention package would have greatest impact in improving maternal and neonatal healthcare services. All provided data will be treated anonymously and with complete confidentiality.

| 1. Could you please tell us about yourself and your family? Such as age, education level, employment status, number of children, pregnancy status… |
| --- |
|  |
| 1. Did you attend antenatal care clinics while you are pregnant? If yes or No, What were the reasons, Where did you get this service from? |
|  |
| 1. Could you please share with us your experience with ANC services? |
|  |
| 1. Where do you usually give birth? Why do you choose that place? |
|  |
| 1. Could you please share with us the birth experience of your youngest baby? |
|  |
| 1. Do you usually seek Postnatal Care Service (PNC)? Where? What are the reasons? |
|  |
| 1. Could you please share with us your experience with PNC services? |
|  |
| 1. In your opinion, how the services in this facility that you sought for both ANC and PNC can be improved? |
|  |
